# Effects of Zanthoxyli Pericarpium Extracts on Ligature-Induced Periodontitis and Alveolar Bone Loss in Rats

**DOI:** 10.3390/antiox14101159

**Published:** 2025-09-24

**Authors:** Jang-Soo Kim, Beom-Rak Choi, Geun-Log Choi, Hye-Rim Park, Jin-Gwan Kwon, Chan-Gon Seo, Jae-Kwang Kim, Sae-Kwang Ku

**Affiliations:** 1 Department of Anatomy and Histology, College of Korean Medicine, Daegu Haany University, Gyeongsan 38610, Republic of Korea; akamjnj@dhu.ac.kr; 2 Nutracore Co., Ltd., Suwon 16514, Republic of Korea; brchoi@nutracore.co.kr (B.-R.C.); hrpark@nutracore.co.kr (H.-R.P.); jgkwon@nutracore.co.kr (J.-G.K.); cgseo@nutracore.co.kr (C.-G.S.); 3 Department of Veterinary Surgery, College of Veterinary Medicine, Kyungpook National University, Daegu 41566, Republic of Korea; 2023001277@knu.ac.kr; 4 Department of Physiology, College of Korean Medicine, Daegu Haany University, Gyeongsan 38610, Republic of Korea

**Keywords:** Zanthoxyli Pericarpium, periodontitis, anti-inflammatory, alveolar bone loss

## Abstract

Zanthoxyli Pericarpium (ZP), the dried pericarp of mature fruits of *Zanthoxylum schinifolium* Siebold and Zucc., has traditionally been used in East Asian medicine for its medicinal properties, but its therapeutic potential in periodontitis has not been elucidated. In the present study, we investigated the effects of ZP on ligature-induced experimental periodontitis (EPD) in male Sprague Dawley rats. Animals were assigned to vehicle control, ligature control, ZP-treated (25, 50, and 100 mg/kg), or indomethacin-treated (5 mg/kg) groups (*n* = 10 per group) and orally administered the respective treatments daily for 10 days after ligature placement. ZP significantly reduced anaerobic bacterial proliferation and inflammatory cell infiltration in gingival tissue. ZP suppressed the production of inflammatory mediators, such as tumor necrosis factor-α and interleukin-1β, in both gingival tissues and lipopolysaccharide-stimulated RAW 264.7 macrophages, through inhibition of the mitogen-activated protein kinase (MAPK) and nuclear factor-κB (NF-κB) signaling pathways. In addition, ZP decreased myeloperoxidase activity and reduced matrix metalloproteinase-8 expression, thereby preserving collagen areas. ZP also restored the receptor activator of NF-κB ligand/osteoprotegerin (RANKL/OPG) balance, leading to a reduction in osteoclast numbers and their occupancy on the alveolar surface, and it effectively ameliorated horizontal alveolar bone loss. Furthermore, ZP exhibited antioxidant effects by lowering malondialdehyde levels and inducible nitric oxide synthase activity in gingival tissues. Statistical analysis was performed using ANOVA followed by a post hoc test, with significance set at *p* < 0.05. These findings indicate that ZP mitigates periodontitis through combined antimicrobial, anti-inflammatory, antioxidant, and anti-resorptive actions, supporting its potential as a therapeutic candidate for periodontitis.

## 1. Introduction

Periodontitis refers to a chronic inflammatory disorder marked by continuous breakdown of tooth-supporting tissues. Its pathogenesis begins with dysbiotic changes in the oral microbiome, leading to the overgrowth of pathogenic anaerobic bacteria. These microorganisms trigger a host immune response, initially recruiting neutrophils to the gingival tissues, followed by the activation of other immune cells, including macrophages and lymphocytes, which amplify the inflammatory process [1]. Excessive production of key inflammatory mediators such as interleukin-1β (IL-1β) and tumor necrosis factor-α (TNF-α) sustains inflammation and promotes osteoclastogenesis through the activation of the receptor activator of the nuclear factor-κB ligand (RANKL)/RANK pathway, thereby contributing to alveolar bone loss [2]. Infiltrating neutrophils further exacerbate periodontal destruction by releasing matrix metalloproteinases (MMPs), which degrade extracellular matrix components, and by producing excessive reactive oxygen species (ROS), resulting in host tissue damage [3].

Epidemiological studies have reported that periodontitis affects more than 40% of adults in the United States and over 1 billion individuals worldwide, making it one of the most prevalent chronic diseases [4,5]. Severe periodontitis is a major public health concerns because it can cause tooth loss and impair mastication, speech, and overall quality of life. Smoking, low socioeconomic status, and diabetes are recognized as key risk factors for periodontitis [6]. Moreover, recent studies have also suggested an association between periodontitis and systemic diseases, including stroke and myocardial infarction [7,8]. Therefore, effective measures for prevention and treatment are needed not only to maintain oral health but also to reduce systemic health risks.

Numerous treatment strategies have been developed to manage periodontitis, ranging from non-surgical mechanical debridement to adjunctive pharmacological therapies [9]. Scaling and root planing (SRP) remains the primary therapy for periodontitis. However, additional use of systemic or locally delivered antimicrobial agents is often required for better clinical outcomes [10,11]. Despite their effectiveness, prolonged systemic antibiotic use may cause adverse effects such as allergic reactions, nephritis and gastrointestinal disturbances [12]. Consequently, natural products have attracted increasing attention as alternative therapies, because of their bioactive compounds with anti-inflammatory, antioxidant, and antimicrobial properties [13]. Clinical trials have demonstrated that natural products such as resveratrol, green tea, and propolis can improve clinical periodontal parameters such as plaque index, bleeding on probing, and probing depth [14]. These findings encourage further exploration of medicinal herbs as safer and more sustainable therapeutic options for managing periodontitis.

According to the Korean Pharmacopoeia, Zanthoxyli Pericarpium (ZP) refers to the dried pericarp of mature fruits of *Zanthoxylum piperitum* De Candolle, *Zanthoxylum bungeanum* Maxim., or *Zanthoxylum schinifolium* Siebold and Zucc. Traditionally, it has been used in East Asian medicine to manage intestinal disorders such as stomach pain, indigestion, and diarrhea [15]. Recent pharmacological studies have reported that ZP extracts exert protective effects against dextran sodium sulfate-induced colitis and carbon tetrachloride-induced hepatotoxicity, suggesting anti-inflammatory and antioxidant properties of ZP [15,16]. Although dysregulated inflammation and excessive oxidative stress are also key mechanisms in the pathogenesis of periodontitis, the efficacy of ZP in this disease has not yet been elucidated. Therefore, the present study investigated the protective effects of ZP (extracted from *Z. schinifolium* Siebold and Zucc.) in a ligature-induced experimental periodontitis (EPD) rat model, focusing on its potential antimicrobial, anti-inflammatory, antioxidant, and bone-protective actions. Based on these properties, we hypothesized that oral administration of ZP would attenuate periodontal inflammation, oxidative stress, and alveolar bone loss. To further explore the underlying molecular mechanisms, we examined its effects in lipopolysaccharide (LPS)-stimulated RAW 264.7 macrophages. By combining both in vivo and in vitro experiments, this study provides novel evidence supporting ZP as a promising candidate for periodontal therapy.

## 2. Materials and Methods

### 2.1. Preparation of Test Materials

Fresh *Z. schinifolium* Siebold and Zucc. was harvested in Jinan-gun, Jeollabuk-do Province, Republic of Korea (35.789° N, 127.427° E). The dried form of the same material was subsequently obtained from a local herbal shop in the same region. A voucher specimen (encoded as ZP2022Ku01) has been deposited in the Herbarium of the Medical Research Center for Herbal Convergence on Liver Disease, Daegu Haany University. In addition, a crude drug reference standard sample was obtained from the National Institute of Food and Drug Safety Evaluation (NIFDS), Ministry of Food and Drug Safety (MFDS), Republic of Korea, and the harvested material was authenticated by comparison with the reference standard using high-performance liquid chromatography (HPLC) fingerprint analysis. ZP extract powder was manufactured and supplied by Nutracore (Suwon, Republic of Korea), as specified in the Supplementary Information (Batch No. ZS-H221108). Syringin was identified in the ZP extract as a marker compound using HPLC analysis (Supplementary Figures S1, S2, and Table S1), and its content was quantified as 1.67 mg/g. For experimental use, ZP was dissolved in distilled water at concentrations of 5, 10, and 20 mg/mL. Indomethacin (IND), used as a reference drug in animal studies, and dexamethasone (DX), used for in vitro experiments, were both obtained from Sigma-Aldrich (St. Louis, MO, USA).

### 2.2. Cell Culture

RAW 264.7 macrophage cells were purchased from the American Type Culture Collection (ATCC; Rockville, MD, USA) and cultured in Dulbecco’s Modified Eagle Medium (DMEM; HyClone Laboratories, Logan, UT, USA) containing 10% fetal bovine serum (FBS; Lonza, Walkersville, MD, USA) and 1% Antibiotic–Antimycotic solution (HyClone Laboratories). Cells were cultured at 37 °C in a humidified atmosphere containing 5% CO_2_.

### 2.3. Measurement of Nitric Oxide (NO) Production

RAW 264.7 cells were treated with ZP or DX in the presence of LPS. After treatment, the conditioned medium was collected and centrifuged. A 100 μL aliquot of the supernatant was mixed with an equal volume of Griess reagent. The absorbance was then measured at 540 nm using a microplate reader, and the results are expressed as fold changes relative to the control group.

### 2.4. Immunoblot Analysis

For whole-cell lysate preparation, cells were lysed in radioimmunoprecipitation assay (RIPA) buffer supplemented with phosphatase and protease inhibitor cocktail (GenDEPOT, Barker, TX, USA) for 1 h on ice. Lysates were centrifuged, and protein concentrations were determined using the BCA assay (Thermo Fisher Scientific, Rockford, IL, USA). Proteins (equal in amount) were electrophoresed using SDS-PAGE, transferred to nitrocellulose membranes, and incubated with primary and HRP-conjugated secondary antibodies. Bands were visualized by chemiluminescence and quantified using ImageJ (version 1.53k; https://imagej.nih.gov/ij, accessed on 12 June 2020).

### 2.5. Animal Husbandry

Sixty male Sprague Dawley rats (6 weeks old) were obtained from OrientBio (Seongnam, Republic of Korea) and housed under controlled environmental conditions (20–25°C, 50–55% humidity, and a 12 h light/dark cycle). After 9 days of acclimatization, sixty rats were allocated into six groups (10 animals per group), organized as follows: (1) Intact vehicle control group (non-ligated, orally administered distilled water), (2) EPD induction group (ligated, orally administered distilled water), (3) IND group (ligated, orally administered IND at 5 mg/kg as a reference drug), ZP25 group (ligated, orally administered ZP at 25 mg/kg), ZP50 group (ligated, orally administered ZP at 50 mg/kg), and ZP100 group (ligated, orally administered ZP at 100 mg/kg).

### 2.6. Induction of EPD

To induce EPD, a sterilized 3–0 nylon ligature (AILEE, Busan, Republic of Korea) was placed around the cervical region of the upper left incisor of each rat. Anesthesia was achieved via inhalation of 2–3% isoflurane (Hana Pharm. Co., Hwasung, Republic of Korea) using a rodent inhalation anesthesia system (Surgivet, Waukesha, WI, USA) with a ventilator (Havard Apparatus, Cambridge, UK). The ligature was tied on the buccal side of the tooth, positioned subgingivally on the palatal side and supragingivally on the buccal side. In the intact vehicle control group, the cervical area of the upper left incisor was merely identified without ligature placement.

### 2.7. Measurement of Body Weights

Body weights were measured once daily, starting from one day prior to ligature placement and continuing throughout the entire experimental period, using an electric balance (Precisa Instruments, Zurich, Switzerland).

### 2.8. Measurement of Alveolar Bone Loss

After sacrifice, the maxillary bone containing the ligature placement site was photographed and then excised. Horizontal bone loss was assessed as the distance from the cusp tip to the alveolar bone along the axis of the upper left incisor root, measured with an electronic digital caliper and expressed in millimeters.

### 2.9. Microbiological Analysis

Buccal gingival tissues adjacent to the ligated upper left incisor were collected and homogenized in 0.3 mL of brain heart infusion (BHI) broth. The homogenates were diluted and plated onto BHI agar containing sodium propionate, lithium chloride, cysteine hydrochloride, and defibrinated sheep blood to support anaerobic growth. Plates were incubated under anaerobic conditions at 37 °C for 48 h, and colony-forming units (CFUs) were counted and are expressed as ×10^2^ CFU/g of tissue. All media and reagents were obtained from Becton, Dickinson and Company (BD; Franklin Lakes, NJ, USA).

### 2.10. Measurement of Myeloperoxidase (MPO) Activity

MPO activity was assessed in gingival tissues collected from the ligature-placement site 11 days after EPD induction, following previously established protocols [17,18]. Briefly, tissues were placed in 0.5% hexadecyltrimethylammonium bromide prepared in 50 mM potassium phosphate buffer (pH 6.0) to solubilize MPO. After homogenization, the homogenates were adjusted with additional buffer to a final volume of 400 μL per 15 mg of tissue and incubated for 12 min. The samples were then centrifuged at 1000× *g* for 12 min at 4 °C, and 100 μL of the resulting supernatant was mixed with 2 mL of phosphate buffer (50 mM, pH 6.0) containing 0.167 mg/mL *o*-dianisidine dihydrochloride and 0.0005% hydrogen peroxide. The absorbance of this solution at 460 nm was then recorded with a UV/Vis spectrophotometer. One unit (U) of MPO activity was defined as the amount of enzyme that catalyzing the degradation of 1 μmol of hydrogen peroxide per min at 25 °C. Results are expressed as U per milligram of tissue (U/mg tissue).

### 2.11. Radical Scavenging Assay

The radical scavenging capacity of ZP was evaluated using the DPPH (2,2-diphenyl-1-picrylhydrazyl) assay. ZP was dissolved in distilled water at final concentrations of 10–300 μg/mL and mixed with 150 μM DPPH solution. After incubation for 30 min at room temperature in the dark, absorbance was measured at 517 nm with a microplate reader. Radical scavenging activity (%) was calculated as [(S − S_0_)/(C − C_0_)] × 100, where S and S_0_ represent the absorbance values of DPPH with and without the sample, and C and C_0_ indicate those of the solvent control.

### 2.12. Measurement of Malondialdehyde (MDA) Levels

Lipid peroxidation within gingival tissues was evaluated by determining MDA with a modified thiobarbituric acid reactive substance (TBARS) assay [18]. Buccal gingival tissues were homogenized in Tris-HCl buffer (50 mM, pH 7.4) containing EGTA and PMSF. The resulting homogenates were incubated with a reaction mixture containing SDS, acetic acid, and thiobarbituric acid at 95 °C for 1 h, and centrifuged. The absorbance of the supernatant was measured at 650 nm, and MDA levels are expressed relative to tissue weight (μM/mg).

### 2.13. Measurement of Inducible Nitric Oxide Synthase (iNOS) Activity

iNOS activity in gingival tissue was assessed by measuring the conversion of L-[^3^H]-arginine to L-[^3^H]-citrulline, following established protocols [18,19]. Tissue homogenates were incubated at 22 °C for 30 min in a reaction mixture containing cofactors such as NADPH, calmodulin, tetrahydrobiopterin, calcium. The enzymatic reaction was stopped with HEPES buffer (pH 5.5) containing EDTA and EGTA. For control assays, reactions were conducted without NADPH (to determine non-enzymatic conversion) and with excess EGTA (assess calcium-independent activity). Reaction mixtures were passed through ion-exchange columns, and L-[^3^H]-citrulline formation was quantified by liquid scintillation counting. Results are expressed as fM/mg/min.

### 2.14. Enzyme-Linked Immunosorbent Assay (ELISA)

ELISA was performed to quantify prostaglandin E_2_ (PGE_2_), MMP-8, TNF-α, and IL-1β, in gingival tissues or culture supernatants using commercially available kits, following the manufacturers’ instructions, as previously described [18].

### 2.15. Reverse Transcription Quantitative Polymerase Chain Reaction (RT-qPCR)

Total RNA was extracted from maxillary gingival tissues and RAW 264.7 cells using commercially available phenol-based reagents according to the manufacturer’s instructions. RNA was reverse-transcribed into cDNA using a commercial reverse transcription kit with oligo (dT) primers. Real-time PCR was performed using diluted cDNA and gene-specific primers on a thermal cycler. Gene expression levels were calculated using the comparative threshold cycle (ΔΔCt) method and normalized to reference genes such as β-actin or GAPDH [20]. Primer sequences used in the experiments are provided in Table 1.

### 2.16. Histopathological Analysis

The maxillary region, encompassing both left and right upper incisors and the ligature placement site, was harvested and preserved in 10% neutral-buffered formalin for fixation. Afterward, the specimens underwent decalcification using formic acid and sodium hydroxide, with daily replacement of the solution for five consecutive days. Samples were then transversely trimmed to include both incisors, embedded in paraffin, and sectioned at 3 to 4 μm thickness using a microtome.

Representative sections were stained with hematoxylin and eosin (H&E) following established protocols [21,22]. Histological evaluation of tissues was performed under light microscopy (Eclipse 80*i*, Nikon, Tokyo) equipped with a digital camera system (ProRes™ C5, Jenoptik Optical Systems GmbH, Jena, Germany) and analyzed blindly using image analyzing software (*i*Solution FL, IMT *i*-solution Inc., Bernaby, BC, Canada). The inter-incisor area at the ligature site was scored from 0 to 3 based on inflammatory cell infiltration and the integrity of alveolar bone and cementum, as described previously [21,22,23]. In addition, histomorphometric analysis was conducted to quantify inflammatory cell infiltration (cells/mm^2^) and collagen area percentage (%/mm^2^) within the gingival tissues adjacent to the left incisor. Alveolar bone volume (%), osteoblast number (cells/mm^2^), and their alveolar bone surface occupancy (OC/BS, %) were also measured within the alveolar bone region between the incisors, excluding the tooth roots. All morphometric assessments utilized the same image analysis system and were performed with blinding to group allocation, in accordance with previously established methodologies [21,22].

### 2.17. Statistical Analysis

Numerical values are expressed as mean ± standard deviation (SD) from at least three independent in vitro experiments or from 10 animals. To assess statistical differences among groups, a one-way analysis of variance test (ANOVA) or Welch’s ANOVA was applied, depending on the homogeneity of variance. Post hoc comparisons were performed using Tukey’s honestly significant difference (HSD) test or Dunnett’s T3 test, as appropriate. A *p*-value of less than 0.05 was considered statistically significant. All statistical analyses were performed using SPSS software (Release 18.0, SPSS Inc., Chicago, IL, USA).

## 3. Results

### 3.1. ZP Suppresses the Expression of Pro-Inflammatory Mediators in LPS-Stimulated RAW 264.7 Macrophages

**Figure 1:**
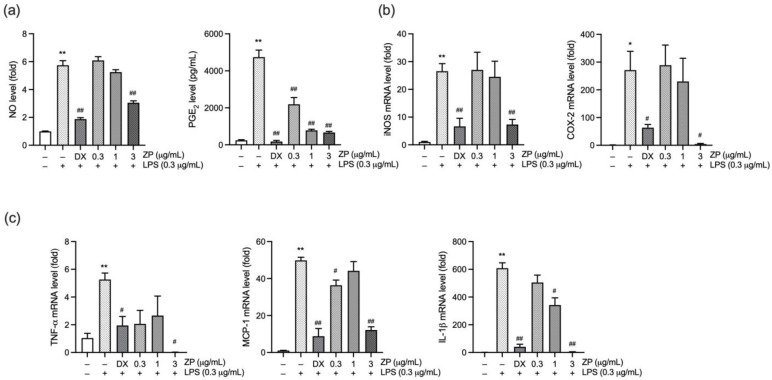
Zanthoxyli Pericarpium (ZP) suppresses the production of inflammatory mediators. RAW 264.7 cells were incubated with dexamethasone (DX; 1 μM) or ZP extract (0.3–3 mg/mL) for 1 h, followed by exposure to lipopolysaccharide (LPS; 0.3 μg/mL) for (**c**) 6 or (**a**,**b**) 18 h. (**a**) Nitric oxide (NO) level and prostaglandin E_2_ (PGE_2_) level in conditioned media. (**b**) inducible nitric oxide synthase (iNOS) (left) and cyclooxygenase-2 (COX-2) (right) mRNA levels. (**c**) mRNA levels of pro-inflammatory cytokines, including tumor necrosis factor-α (TNF-α), monocyte chemoattractant protein-1 (MCP-1), and interleukin-1β (IL-1β), were determined by RT-qPCR. Relative expression was calculated using glyceraldehyde-3-phosphate dehydrogenase as the internal control. Significance versus vehicle-treated group, * *p* < 0.05, ** *p* < 0.01; versus LPS-treated group, ^#^ *p* < 0.05, ^##^ *p* < 0.01.

To investigate the potential anti-inflammatory role of ZP, we examined its influence on the production of inflammatory mediators in LPS-stimulated RAW 264.7 macrophages. Before assessing its anti-inflammatory activity, we evaluated whether ZP affected the viability of HaCaT (human keratinocytes), HDFn (neonatal human dermal fibroblasts), and RAW 264.7 (murine macrophage) cells. No significant reduction in cell viability was observed in any of the cell types following treatment with ZP (0.001–10 mg/mL) (Supplementary Figure S3). Next, cells were pre-incubated with ZP (0.3–3 mg/mL) or DX (1 μM) for 1 h and further exposed to LPS (0.3 μg/mL). After 18 h of incubation, levels of NO and PGE_2_ were measured in the culture supernatant. As expected, LPS significantly elevated NO and PGE_2_ levels compared to the vehicle-treated group. However, ZP treatment suppressed both NO and PGE_2_ levels in a dose-dependent manner, with a significant reduction at the highest concentration (3 mg/mL) (Figure 1a). In addition, we assessed the mRNA expression of iNOS and COX-2, which are responsible enzymes for NO and PGE_2_ synthesis, respectively. LPS markedly upregulated both iNOS and COX-2 mRNA levels compared to the vehicle-treated group, whereas 3 mg/mL of ZP significantly reduced their expression (Figure 1b). In a separate experiment, the expression of pro-inflammatory cytokines, including TNF-α, monocyte chemoattractant protein-1 (MCP-1), and IL-1β, was evaluated after 6 h of LPS exposure. Pretreatment with 3 mg/mL of ZP significantly downregulated the mRNA levels of all three cytokines (Figure 1c). These findings suggest that ZP mitigates LPS-induced inflammatory responses by inhibiting the production of inflammatory mediators.

### 3.2. ZP Suppresses LPS-Induced Inflammatory Response by Inhibiting Mitogen-Activated Protein Kinase (MAPK) and NF-κB Signaling Pathways

**Figure 2:**
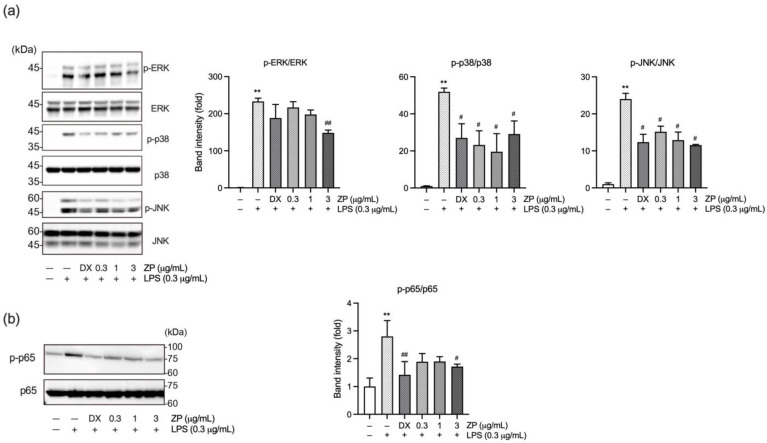
ZP attenuates LPS-induced inflammation through suppression of mitogen-activated protein kinase (MAPK) and nuclear factor-κB (NF-κB) pathways. RAW 264.7 macrophages were incubated with DX (1 μM) or ZP (0.3–3 mg/mL) for 1 h and subsequently stimulated with 0.3 μg/mL of LPS (0.3 μg/mL) for 30 min. Phosphorylated and total forms of (**a**) MAPKs and (**b**) p65 were examined by immunoblotting, with the intensity of each phosphorylated band normalized to its corresponding total protein. Significance versus vehicle-treated group, ** *p* < 0.01; versus LPS-treated group, ^#^ *p* < 0.05, ^##^ *p* < 0.01.

To clarify how ZP exerts anti-inflammatory activity, its influence on MAPK and NF-κB signaling in LPS-stimulated RAW 264.7 cells was investigated. RAW 264.7 cells were exposed to ZP (0.3–3 mg/mL) or DX (1 μM) for 1 h and were then stimulated with LPS (0.3 μg/mL). After 30 min of incubation, protein lysates were analyzed by immunoblotting to assess the phosphorylation status of extracellular signal-regulated kinase (ERK), p38, c-Jun N-terminal kinase (JNK), and p65. As expected, LPS markedly increased phosphorylation of ERK, p38, and JNK compared to the vehicle-treated group. ZP pretreatment attenuated LPS-induced MAPK phosphorylation. In particular, ERK phosphorylation was significantly reduced at 3 mg/mL of ZP, while p38 and JNK phosphorylation were significantly inhibited at all tested concentrations (0.3–3 mg/mL) (Figure 2a). Next, we assessed p65 phosphorylation as an indicator of NF-κB pathway activation, reflecting its transcriptional activity [24]. Similarly, LPS-induced phosphorylation of p65 was decreased by ZP, with a significant reduction at 3 mg/mL (Figure 2b). These results suggest that ZP suppresses LPS-induced inflammatory responses by inhibiting the activation of NF-κB signaling pathways.

### 3.3. Experimental Schedule and Changes in Body Weight

**Figure 3:**
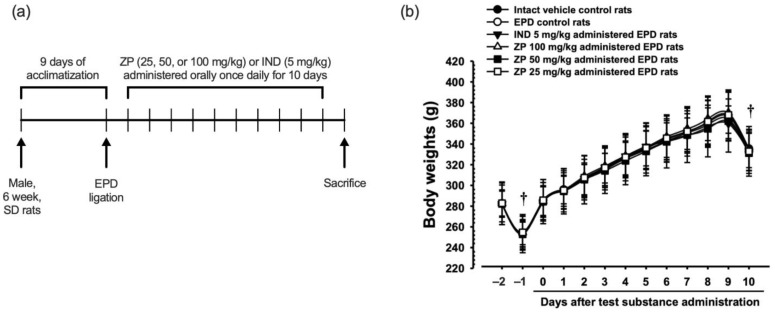
Experimental schedule and changes in body weight. (**a**) Scheme of animal experiment. (**b**) Body weights were recorded daily, beginning one day prior to ligature placement and continuing throughout the entire experimental period. All values are expressed mean ± SD of ten rats. ^†^ All animals were fasted overnight prior to both ligature placement and sacrifice.

24 h after ligature placement, test substances including IND (5 mg/kg) or ZP (25, 50, and 100 mg/kg) were orally administered once daily for 10 days, while control groups were given distilled water. Animals were sacrificed 24 h after the final administration (Figure 3a).

Body weights of the animals were measured daily, beginning one day prior to ligature placement and continuing throughout the 11 days of experimental period. No significant differences in body weight were observed between the EPD control and intact vehicle control groups. Similarly, administration of ZP (25, 50, or 100 mg/kg), as well as IND treatment, was not associated with significant alterations in body weight compared with the EPD control group (Figure 3b).

### 3.4. ZP Attenuates Alveolar Bone Loss in EPD Rats

**Figure 4:**
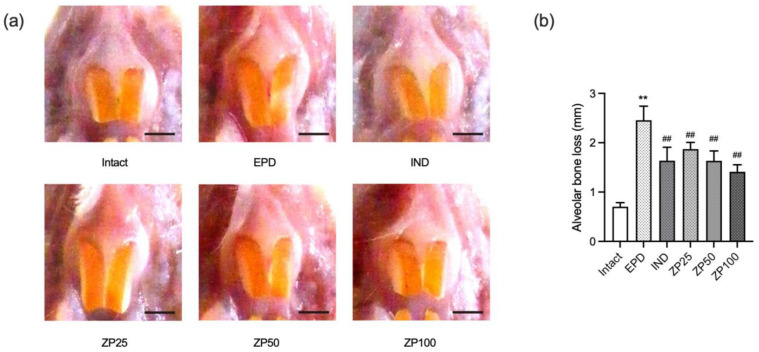
ZP attenuates alveolar bone loss in EPD rats. (**a**) Representative images of the upper left incisor region showing the ligature placement site. Scale bars = 3.00 mm. (**b**) Quantitative analysis of horizontal alveolar bone loss, determined as the cusp tip to bone surface distance, was measured along the axis of the upper left incisor. Data are presented as mean ± SD from ten animals per group. Significance versus intact vehicle control group, ** *p* < 0.01; versus EPD control group, ^##^ *p* < 0.01.

To evaluate the effects of ZP on EPD, horizontal alveolar bone loss was measured as a key indicator of tissue destruction. Ligature placement in the EPD control group significantly increased alveolar bone loss compared to the intact vehicle control group. Treatment with IND significantly attenuated this alveolar bone loss. Similarly, oral administration of ZP (25, 50, and 100 mg/kg) significantly and dose-dependently reduced alveolar bone loss (Figure 4).

### 3.5. ZP Decreased the Gingival Anaerobic Bacterial Count and MPO Activity

**Figure 5:**
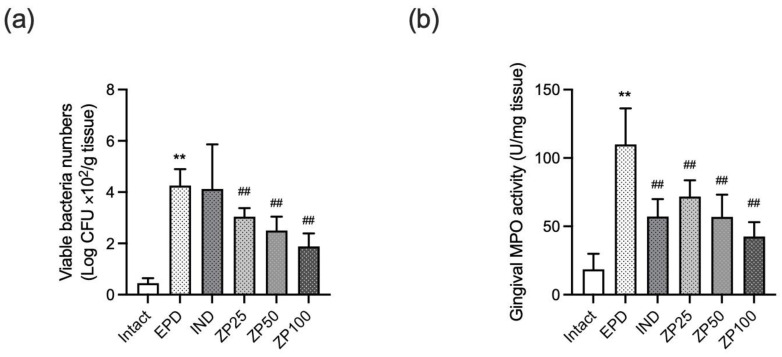
Effect of ZP on total anaerobic bacterial counts and myeloperoxidase (MPO) activity in buccal gingival tissues. (**a**) Anaerobic bacterial count. Gingival tissues were homogenized, and cultured under anaerobic conditions, and colonies counted as ×10^2^ colony-forming units (CFU)/g of tissue. (**b**) MPO activity. One unit is defined as the amount of enzyme degrading 1 μM H_2_O_2_ per min at 25 °C. Data are presented as mean ± SD from ten animals per group. Significance versus intact vehicle control group, ** *p* < 0.01; versus EPD control group, ^##^ *p* < 0.01.

Ligature placement in the EPD control group led to a significant increase in anaerobic bacterial counts within gingival tissue. Oral administration of ZP (25, 50, and 100 mg/kg) significantly and dose-dependently reduced this bacterial overgrowth (Figure 5a). Furthermore, to evaluate neutrophil infiltration, MPO activity was assessed in gingival tissue. Ligature placement in the EPD control group markedly elevated MPO activity compared to the intact vehicle control group. While IND treatment significantly suppressed this increase, oral administration of ZP (25, 50, and 100 mg/kg) also significantly and dose-dependently decreased MPO activity (Figure 5b).

### 3.6. ZP Reduced the Gingival Expression of Pro-Inflammatory Mediators in EPD Rats

**Figure 6:**
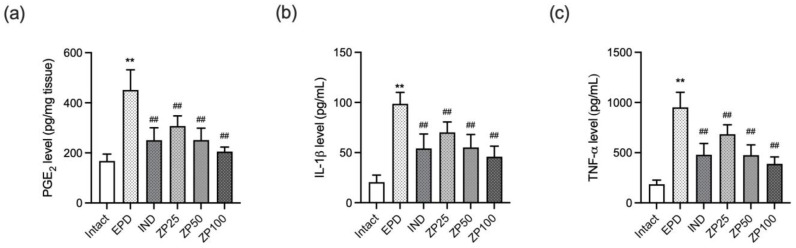
ZP lowered the gingival levels of pro-inflammatory mediators including (**a**) PGE_2_, (**b**) IL-1β, and (**c**) TNF-α in EPD rats. Data are presented as mean ± SD from ten animals per group. Significance versus intact vehicle control group, ** *p* < 0.01; versus EPD control group, ^##^ *p* < 0.01.

To elucidate the anti-inflammatory effects of ZP, the gingival concentrations of PGE_2_, IL-1β, and TNF-α were measured by ELISA. Ligature placement in the EPD control group significantly increased the expression of these mediators compared to the intact vehicle control group. While IND treatment significantly reduced their expression, oral administration of ZP (25, 50, and 100 mg/kg) also significantly and dose-dependently suppressed the elevated levels of PGE_2_, IL-1β, and TNF-α (Figure 6).

### 3.7. ZP Exhibits Antioxidant Effects by Reducing Free Radicals, Lipid Peroxidation, and Nitrosative Stress

**Figure 7:**
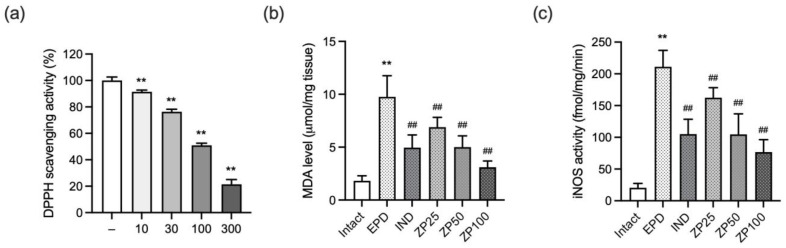
ZP exhibits antioxidant effects by reducing free radicals, lipid peroxidation, and nitrosative stress. (**a**) Radical scavenging activity was assessed by the DPPH assay. (**b**) Malondialdehyde (MDA) levels and (**c**) iNOS activity were measured in gingival tissues. Data (**b**,**c**) are presented as mean ± SD from ten animals per group. Significance versus vehicle-treated or intact vehicle control group, ** *p* < 0.01; versus EPD control group, ^##^ *p* < 0.01.

To verify the antioxidant effects of ZP, we first evaluated its free radical scavenging activity using a DPPH assay. ZP exhibited significant radical scavenging activity at concentrations ranging from 10 to 300 μg/mL (Figure 7a). Next, to assess the effect of ZP on lipid peroxidation, MDA levels in gingival tissues were measured. Ligature placement significantly elevated MDA levels, which were markedly and dose-dependently reduced by oral administration of ZP (25, 50, and 100 mg/kg) (Figure 7b). In addition, to investigate the impact of ZP on nitrosative stress, iNOS activity in gingival tissues was measured [25]. Ligature placement significantly increased iNOS activity, which was also significantly and dose-dependently attenuated by ZP treatment at all tested doses (Figure 7c).

### 3.8. ZP Suppresses the Expression of MMP-8 in Gingival Tissues of EPD Rats

**Figure 8:**
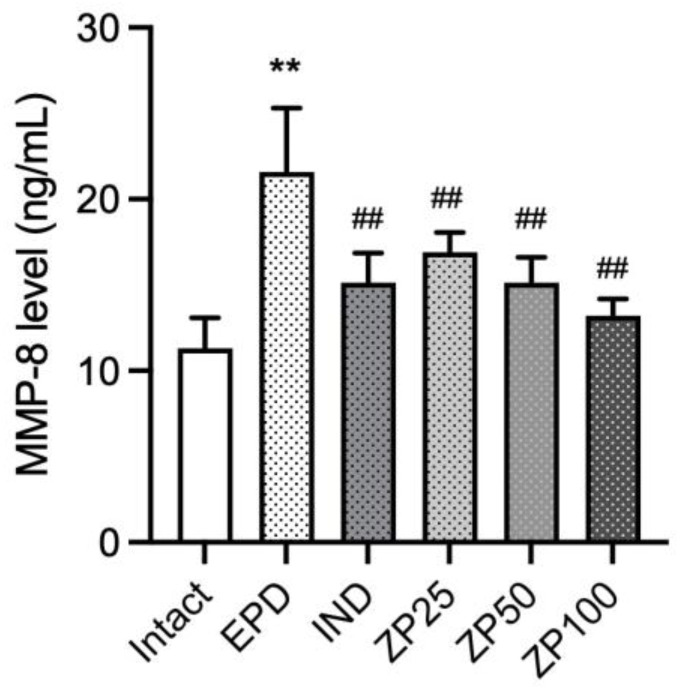
ZP suppresses matrix metalloproteinase-8 (MMP-8) levels in gingival tissues of EPD rats. Data are presented as mean ± SD from ten animals per group. Significance versus intact vehicle control group, ** *p* < 0.01; versus EPD control group, ^##^ *p* < 0.01.

To assess the effects of ZP on the tissue-destructive enzyme activity in periodontitis, we examined the expression of MMP-8. MMP-8 levels were significantly increased following ligature placement in the EPD control group. Notably, treatment with ZP (25, 50, and 100 mg/kg) led to a significant and dose-dependent reduction in MMP-8 expression (Figure 8).

### 3.9. ZP Modulates Bone Remodeling in Periodontitis by Regulating the RANKL/Osteoprotegerin (OPG) Axis

**Figure 9:**
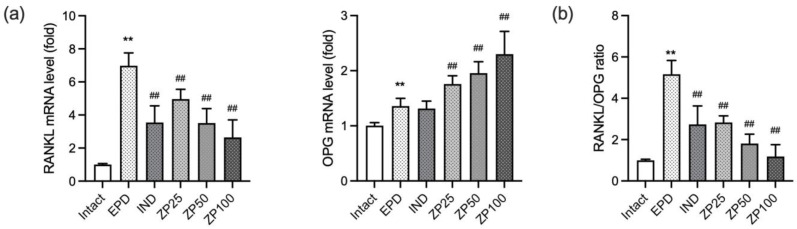
ZP modulates mRNA expressions of (**a**) receptor activator of NF-κB ligand (RANKL), osteoprotegerin (OPG), and (**b**) RANKL/OPG ratio in gingival tissue of EPD rats. Relative mRNA levels were quantified by RT-qPCR and normalized to β-actin. The ratio was calculated by dividing the normalized RANKL expression by that of OPG. Data are presented as mean ± SD from ten animals per group. Significance versus intact vehicle control group, ** *p* < 0.01; versus EPD control group, ^##^ *p* < 0.01.

In the EPD control group, ligature placement significantly increased RANKL mRNA expression while decreasing OPG mRNA expression. However, oral administration of ZP (25, 50, and 100 mg/kg) significantly and dose-dependently downregulated RANKL expression and upregulated OPG mRNA expression (Figure 9a). As a result, the elevated RANKL/OPG ratio observed in the EPD control group was significantly and dose-dependently reduced by ZP treatment (Figure 9b).

### 3.10. Histopathological Changes in Maxillary Regions

**Figure 10:**
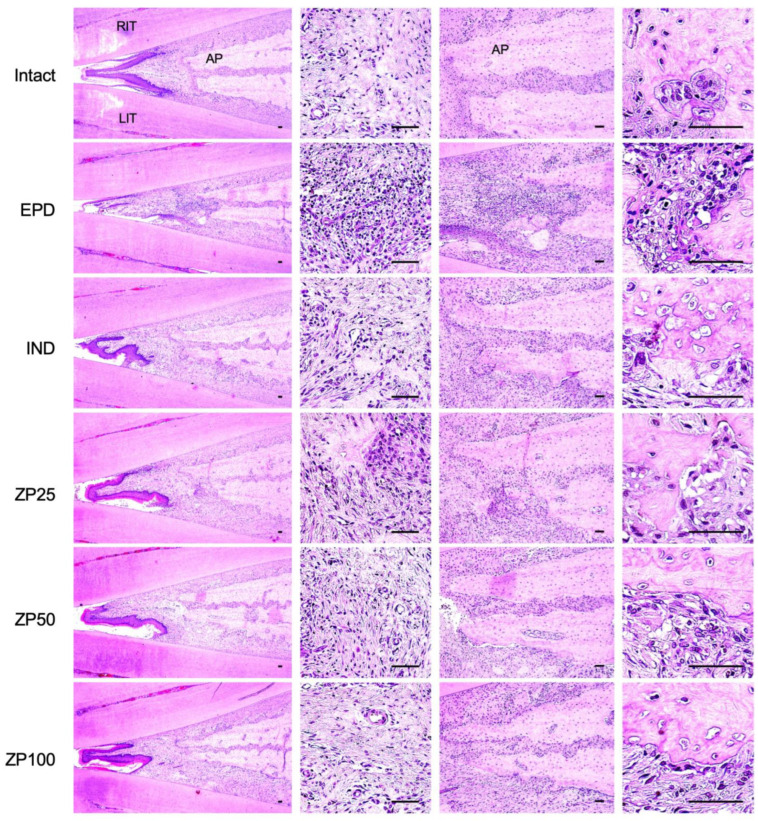
Hematoxylin and eosin-stained sections of gingival tissue and alveolar bone areas from the region between the upper incisors. Scale bar = 50 μm. LIT, left incisor tooth; RIT, right incisor tooth; AP, alveolar process.

Histological observations were performed on the maxillary regions surrounding the ligature placement site, including both gingival tissue and alveolar bone area (Figure 10). In gingival tissue, histological scores were determined based on a previously reported method assessing inflammatory cell infiltration, and the integrity of alveolar cementum and bone, [21,22,23]. Histological scores were significantly elevated by ligature placement in the EPD control group. Treatment with IND significantly reduced these scores. Similarly, oral administration of ZP (25, 50, and 100 mg/kg) significantly and dose-dependently decreased the histological scores. In addition, ZP treatment effectively attenuated key. pathological features of periodontitis, including inflammatory cell infiltration (cells/mm^2^) and collagen fiber degradation (%/mm^2^) (Table 2).

In the alveolar region, histomorphometric analysis was also conducted to evaluate the effect of ZP on bone resorption. Parameters including alveolar bone volume (%), number of osteoclasts (cells/mm^2^), and their alveolar bone surface occupancy (OC/BS, %/mm^2^) were assessed. As expected, ligature placement in the EPD control group significantly reduced alveolar bone volume while significantly increasing both osteoclast numbers and their occupied area on the alveolar bone surface, indicating enhanced osteoclast activity and bone resorption. However, oral administration of ZP (25, 50, and 100 mg/kg) significantly and dose-dependently reversed these changes, suggesting a protective effect against alveolar bone loss (Table 3).

## 4. Discussion

Periodontitis is a prevalent disease characterized by the loss of gingival tissue, periodontal ligament, cementum and alveolar bone. Experimental animal models have enabled the investigation of its pathogenesis and the development of novel therapeutic strategies. Among these, the EPD model in rats is widely utilized due to its rapid onset, consistent bone loss, and translational relevance [26]. Thread ligature placement around the teeth promotes bacterial accumulation in periodontal tissues, leading to inflammation, loss of periodontal attachment, and alveolar bone resorption [18,21,22]. In the present study, we employed the upper incisor ligation model to evaluate the preventive and therapeutic potential of ZP against periodontitis. Compared with the conventional molar ligation model, the incisor model has the limitation of reduced comparability with most of the existing literature, as the molar approach is more commonly used. Nevertheless, it offers advantages as better visibility, procedural ease, and a moderate phenotype, which make it suitable for evaluating the preventive and therapeutic effects of natural products [27]. Therefore, we used this incisor model to comprehensively evaluate the therapeutic effects of ZP, focusing on its anti-inflammatory, antimicrobial, antioxidant, and bone-protective actions.

Periodontitis primarily develops from a dysbiosis of the oral microbiota, resulting in the overgrowth of pathogenic bacteria and chronic inflammation. Oral anaerobic bacteria, including *Porphyromonas gingivalis*, *Treponema denticola*, and *Tannerella forsythia*, have traditionally been regarded as a major etiological group in periodontitis, due to their virulence and ability to form biofilms [1]. In the present study, ligature placement significantly elevated anaerobic bacterial counts, indicating dysbiosis of the microbiota. Remarkably, oral administration of ZP significantly reduced these bacterial counts in a dose-dependent manner (Figure 5a). However, the bacterial profiles observed in the ligature-induced periodontitis model in rats are dominated by *Streptococcus* and *Actinomyces* species, which differ from the complex microbiota associated with human periodontitis [28]. Therefore, further studies are essential to evaluate the antimicrobial effects of ZP against key human periodontal pathogens to enhance translational relevance.

Neutrophils are key responders in gingival inflammation, migrating to infection sites to eliminate pathogens. The polymorphonuclear neutrophil (PMN), which accounts for 50–70% of the total leukocyte infiltrate, acts as the first line of defense against bacterial pathogens, employing mechanisms such as degranulation, chemotaxis, phagocytosis, and the release of ROS [29]. However, in periodontitis, their persistent activation and accumulation lead to the release of destructive enzymes such as MMP-8 and ROS, which damage periodontal tissues, rather than protect periodontal tissues [30,31]. A clinical study also showed that neutrophil counts correlate with disease severity [32]. In the present study, ligature placement increased MPO activity, whereas ZP administration significantly reduced MPO activity in gingival tissue, indicating decreased neutrophil infiltration (Figure 5b).

Pathogenic materials released from microbial biofilms, such as endotoxins, trigger inflammatory and immune responses in gingival tissue. If persistent, these responses can evolve into chronic inflammation, ultimately leading to gingival tissue destruction. This chronic inflammatory state is primarily driven by cytokines or mediators including TNF-α, IL-1β, and PGE_2_, which are upregulated in both human periodontitis and EPD models. In addition to the destruction of gingival tissue resulting directly from inflammation, pro-inflammatory cytokines are strongly associated with bone and attachment loss. In particular, IL-1β and TNF-α are the most extensively studied and are strongly correlated with the development of periodontal disease. IL-1β increases the expression of collagenolytic enzymes and upregulates RANKL, thereby stimulating osteoclastogenesis [33]. Likewise, TNF-α plays a central role in host-mediated tissue damage by recruiting PMNs and stimulating osteoclast formation [34]. Conversely, knockout of TNF-α has been reported to mitigate inflammation and alveolar bone destruction in periodontitis models. [35]. Additionally, exogenous application of PGE_2_ has been shown to induce alveolar bone resorption by stimulating osteoclast activation and proliferation, thereby accelerating periodontal tissue destruction in rat models [36]. In our study, ZP treatment reduced TNF-α, IL-1β, and PGE_2_ in both gingival tissue and macrophages (Figure 1 and Figure 6), suggesting its protective effect against tissue destruction through downregulation of pro-inflammatory mediators.

To explore the underlying mechanism of ZP’s anti-inflammatory action, we investigated its effect on key signaling pathways, particularly MAPKs and NF-κB, both of which are critically involved in inflammatory gene expression. ZP pretreatment inhibited the phosphorylation of ERK, JNK, and p38 MAPKs, as well as phosphorylation of the NF-κB subunit p65 in LPS-stimulated RAW 264.7 cells (Figure 2). These pathways are well-established regulators of cytokine expression in response to bacterial stimuli in periodontal tissue. Notably, oral administration of a p38α inhibitor has been shown to significantly reduce inflammation and cytokine expression, thereby decreasing periodontal bone loss. Conversely, MAPK phosphatase-1 (MKP-1)-deficient mice, which exhibit sustained activation of p38, showed increased osteoclast formation and greater bone loss [37]. Furthermore, the immunohistochemical analysis of gingival tissue from patients with chronic periodontitis demonstrated that p65 immunoreactivity correlates with clinical periodontal scores [38]. These findings suggest that targeting the MAPK and NF-κB signaling pathways could be an effective strategy for controlling inflammation and preventing periodontal tissue destruction.

One hallmark of periodontitis is the degradation of the periodontal connective tissue matrix, particularly type I collagen, which provides structural support. This process begins in gingivitis and progresses during periodontitis, mainly through MMPs [39,40]. Among them, MMP-8, a PMN-derived collagenase, plays a pivot role in the breakdown of type I collagen and has been strongly implicated in periodontal tissue destruction [31]. The activity of neutrophil collagenase detected in the gingival crevicular fluid correlates positively with disease severity, with MMP-8 accounting for most of the activity [41]. Additionally, elevated salivary MMP-8 levels have been reported in patients with periodontitis, showing significant correlations with clinical disease parameters [42]. In our study, ZP administration significantly reduced MMP-8 expression in the gingiva, accompanied by downregulation of MPO activity (Figure 7b and Figure 8). Histopathological analysis further revealed that collagen fiber-occupied regions in gingival tissue, which were diminished by ligature placement, were restored by ZP administration (Table 1).

Alveolar bone loss is the most serious complication of periodontitis and the primary cause of tooth loss, primarily resulting from osteoclast activation. This process is regulated by the balance between RANKL, which promotes osteoclastogenesis, and OPG, which acts as a decoy receptor to inhibit RANKL signaling. An elevated RANKL/OPG ratio is closely associated with increased bone resorption in periodontitis [2]. In our study, ligature placement increased RANKL mRNA expression, reduced OPG expression, and elevated RANKL/OPG ratio, whereas ZP administration restored this balance, indicating anti-bone resorptive effects (Figure 9). Histological evaluation further showed that ligature placement reduced alveolar bone volume, increased both osteoclast numbers and their occupancy on the alveolar bone surface, while ZP administration ameliorated these changes, thereby preserving alveolar bone (Figure 4 and Table 2). Nevertheless, further mechanistic studies using osteoclast models, such as RANKL-induced macrophage differentiation, will be needed to clarify whether the observed effects of ZP are mediated through key pathways involved in osteoclastogenesis, including MAPK and NF-κB inhibition.

Oxidative stress plays a critical role in the pathogenesis of periodontitis. Under physiological conditions, ROS are produced by phagocytic cells, such as neutrophils and macrophages, during pathogen elimination in the gingival tissues. These ROS contribute to host defense by directly killing microorganisms and regulating immune signaling. However, excessive ROS production causes cytotoxic damages to host cells including DNA and proteins through peroxidation and oxidation. This oxidative stress further amplifies inflammation, promotes the release of MMPs, and stimulates osteoclastogenesis, thereby contributing periodontal tissue destruction [43]. Clinical evidence indicates that lipid peroxidation, reflected by elevated MDA levels in gingival crevicular fluid and saliva, is closely associated with the severity of periodontitis [44,45]. For this reason, antioxidants have been explored as adjunctive agents in periodontal disease, showing potential effects in reducing oxidative damage [46]. ZP showed antioxidant potential, as evidenced by dose-dependent radical scavenging activity and reductions in gingival MDA levels and iNOS activity in EPD rats, suggesting mitigation of periodontitis via inhibition of oxidative stress.

## 5. Conclusions

In summary, ZP inhibited key pathological features of periodontitis, including bacterial overgrowth, inflammatory mediator production, oxidative stress, and bone resorption. These findings provide potential clinical relevance and support its development as a putative therapeutic and preventive candidate for periodontal disease. With further evaluation of pharmacokinetic and toxicity to establish appropriate dosing in humans, future clinical studies will be necessary to confirm applicability and therapeutic value of ZP.

## Figures and Tables

**Table 1:** Primer sequences used for RT-qPCR.

Target Gene	Orientation	Sequences (5′⟶3′)	NCBI Accession No.
RANKL	Sense	CTGATGAAAGGAGGGAGCAC,	NM_057149
	Antisense	GAAGGGTTGGACACCTGAATGC	
OPG	Sense	TCCTGGCACCTACCTAAAACAGCA,	U94330
	Antisense	ACACTGGGCTGCAATACACA	
β-actin	Sense	TCAGGTCATCACTATCGCCAAT,	NM_017008
	Antisense	AAAGAAAGGGTGTAAAACGCA	
iNOS	Sense	GACAAGCTGCATGTGACATC,	NM_001313922.1
	Antisense	GCTGGTAGGTTCCTGTTGTT	
COX-2	Sense	TCCAGATCACATTTGATTGA,	NM_011198.5
	Antisense	TCTTTGACTGTGGGAGGATA	
TNF-α	Sense	ATGAGCACAGAAAGCATGAT,	NM_013693.3
	Antisense	TACAGGCTTGTCACTCGAAT	
IL-1β	Sense	ATGGCAACTGTTCCTGAACT,	NM_008361.4
	Antisense	CAGGACAGGTATAGATTCTT	
MCP-1	Sense	TGATCCCAATGAGTAGGCTGG,	NM_011333.3
	Antisense	ATGTCTGGACCCATTCCTTCT	
GAPDH	Sense	AACGACCCCTTCATTGAC,	NM_001411843.1
	Antisense	TCCACGACATACTCAGCAC	

COX-2, cyclooxygenase-2; GAPDH, glyceraldehyde-3-phosphate dehydrogenase; IL-1β, interleukin-1β; iNOS, inducible nitric oxide synthase; MCP-1, monocyte chemoattractant protein-1; OPG, osteoprotegerin; RANKL, receptor activator of nuclear factor-κB ligand; TNF-α, tumor necrosis factor-α.

**Table 2:** Histological scoring and histomorphometric analysis of gingival tissues in the maxillary region around the ligature placement site.

Group	In Gingival Tissues		
	Histological Scores (Max = 3)	Inflammatory Cells (Cells/mm^2^)	Collagen Fibers (%/mm^2^)
Intact vehicle	0.40 ± 0.52	55.80 ± 16.29	77.42 ± 10.75
EPD	2.80 ± 0.42 **	734.60 ± 68.72 **	14.82 ± 3.74 **
IND	1.60 ± 0.52 ^##^	262.80 ± 89.03 ^##^	50.42 ± 7.83 ^##^
ZP (25 mg/kg)	2.00 ± 0.47 ^#^	377.60 ± 114.03 ^##^	34.93 ± 6.91 ^##^
ZP (50 mg/kg)	1.60 ± 0.52 ^##^	265.80 ± 99.82 ^##^	50.11 ± 7.96 ^##^
ZP (100 mg/kg)	1.00 ± 0.47 ^##^	115.60 ± 35.54 ^##^	60.85 ± 7.42 ^##^

Data are presented as mean ± SD from ten animals per group. Significance versus intact control group, ** *p* < 0.01; versus EPD control group, ^#^ *p* < 0.05, ^##^ *p* < 0.01.

**Table 3:** Histological scoring and histomorphometric analysis of alveolar bone regions in the maxillary region around the ligature placement site.

Group	In Alveolar Bone Regions		
	Alveolar Bone Volume (%)	Osteoclast (Cells/mm^2^)	OC/BS (%)
Intact vehicle	78.08 ± 6.55	7.40 ± 2.84	3.73 ± 1.46
EPD	21.34 ± 6.16 **	50.40 ± 7.88 **	67.47 ± 7.01 **
IND	52.37 ± 7.49 ^##^	27.20 ± 5.90 ^##^	30.41 ± 9.20 ^##^
ZP (25 mg/kg)	39.62 ± 8.18 ^##^	35.20 ± 3.43 ^##^	45.33 ± 5.96 ^##^
ZP (50 mg/kg)	52.61 ± 8.98 ^##^	27.60 ± 5.32 ^##^	30.11 ± 8.76 ^##^
ZP (100 mg/kg)	64.34 ± 10.21 ^##^	16.20 ± 3.33 ^##^	17.64 ± 5.72 ^##^

Data are presented as mean ± SD from ten animals per group. Significance versus intact control group, ** *p* < 0.01; versus EPD control group, ^##^ *p* < 0.01.

## Data Availability

Data is contained within the article or Supplementary Material.

## Supplementary Materials

The following supporting information can be downloaded at: https://www.mdpi.com/article/10.3390/antiox14101159/s1, Table S1: HPLC conditions for the analysis of syringin in ZP extract; Figure S1: Flow chart for manufacturing Zanthoxyli Pericarpium (ZP) hot water extracts. Figure S2: Identification of syringin in ZP extract using HPLC; Figure S3: Effects of ZP on cell viability.
