# Supplementary material for: Effects of Zanthoxyli Pericarpium Extracts on Ligature-Induced Periodontitis and Alveolar Bone Loss in Rats
Source: Antioxidants (Basel). 2025 Sep 24;14(10):1159. doi: 10.3390/antiox14101159 (PMC12562097; doi:10.3390/antiox14101159)
Supplement: Supplementary file 1 [file antioxidants-14-01159-s001.zip › antioxidants-3856838-supplementary.pdf]

Supplementary Information

Effects of Zanthoxyli Pericarpium Extracts on Ligature-induced Periodontitis and Alveolar Bone Loss in Rats

Jang-Soo Kim [1,#], Beom-Rak Choi [2,#], Geun-Log Choi [3], Hye-Rim Park [2], Jin-Gwan Kwon [2], Chan-Gon Seo [2], Jae-Kwang Kim [4,*] and Sae-Kwang Ku [1,*]

[1] Department of Anatomy and Histology, College of Korean Medicine, Daegu Haany University, Gyeongsan 38610, Republic of Korea; akamjnj@dhu.ac.kr (J.-S.K.); gucci200@dhu.ac.kr (S.-K.K.)
[2] Nutracore Co., Ltd., Suwon 16514, Republic of Korea; brchoi@nutracore.co.kr (B.-R.C); hrpark@nutracore.co.kr (H.-R.P.); jgkwon@nutracore.co.kr (J.-G.K.); cgseo@nutracore.co.kr (C.-G.S.)
[3] Department of Veterinary Surgery, College of Veterinary Medicine, Kyungpook National University, Daegu 41566, Republic of Korea; 2023001277@knu.ac.kr (G.-L.C.)
[4] Department of Physiology, College of Korean Medicine, Daegu Haany University, Gyeongsan 38610, Republic of Korea; kim-jk@dhu.ac.kr
* Correspondence: kim-jk@dhu.ac.kr (J.-K.K.); gucci200@dhu.ac.kr (S.-K.K.)
[#] These authors contributed equally to this work.

1. Preparation of Zanthoxyli Pericarpium(ZP) Extract

The raw material was extracted with hot water and filtered through a 1 μm membrane. The filtrate was then concentrated and dried using a spray dryer (Fig. S1). For quality control, syringin was identified in the extract by high-performance liquid chromatography (HPLC) analysis. The HPLC conditions are summarized in Table S1. In the chromatogram, a peak from the ZP sample solution corresponded to the syringin standard at a retention time of 40.8 min. Quantitative analysis of the peak area indicated that the ZP extract contained 1.67 mg/g of syringin (Fig. S2).

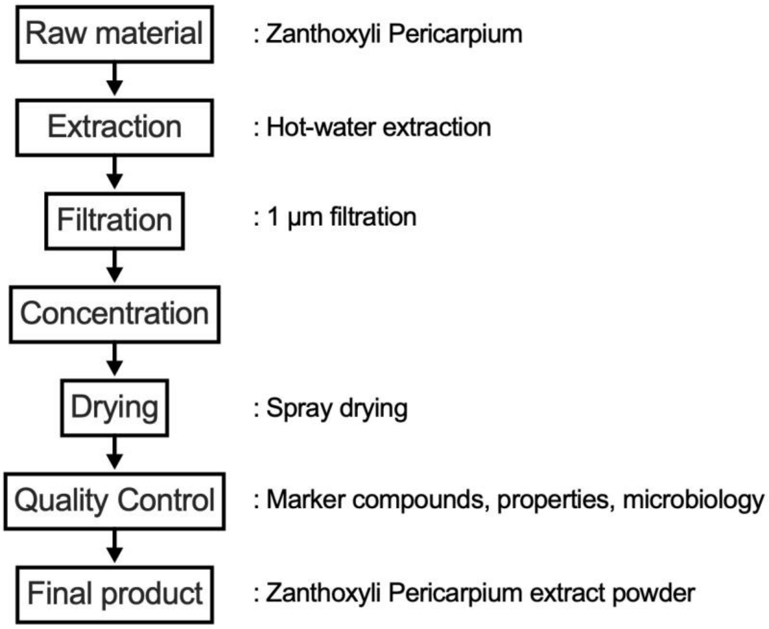

Figure S1. Flow chart for manufacturing Zanthoxyli Pericarpium (ZP) hot water extracts.

Table S1. HPLC conditions for the analysis of syringin in ZP extract.

Parameter	Conditions		
Chromatography system	Agilent 1260 Infinity II		
Detector	UV (265 nm)		
Column	CAPCELL PAK C18 UG120, (4.6 × 250 mm, 5 µm)		
Mobile Phase	A: Water with 0.05% TFA<br>B: Acetonitrile		
Elution program	Time (min)	A (%)	B (%)
	0	95	5
	40	91	9
	50	91	9
	51	5	95
	65	5	95
Flow rate	0.5 mL/min		
Injection volume	5 µL		
Column temperature	30°C		

(a)

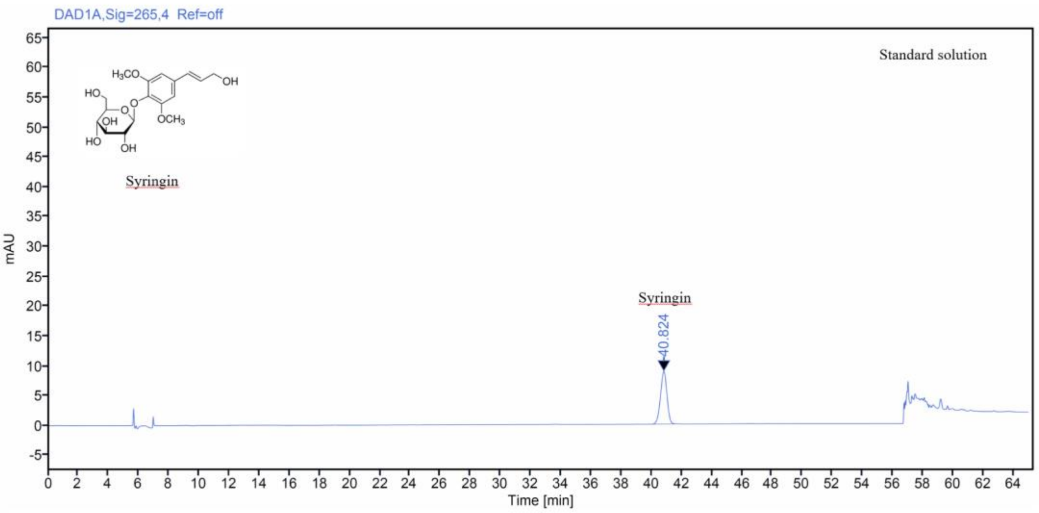

(b)

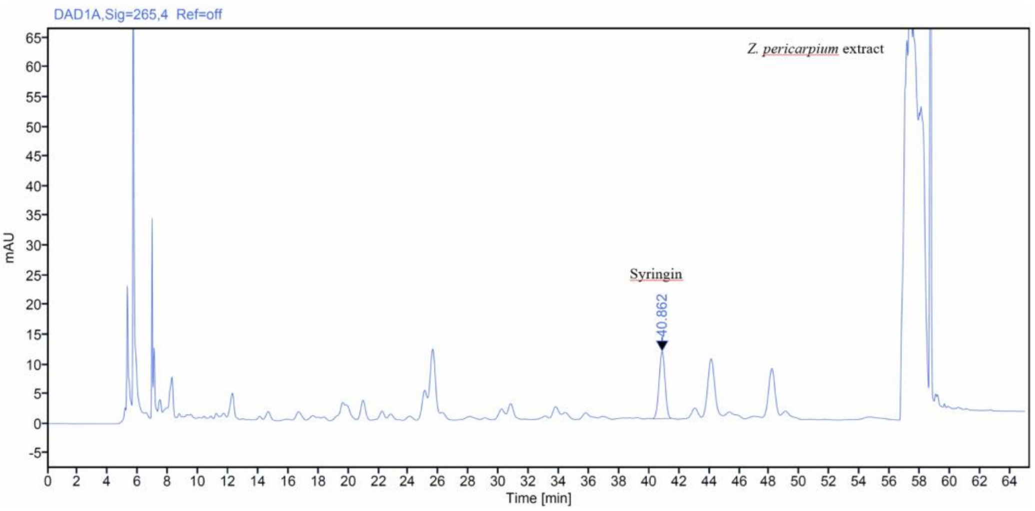

Figure S2. Identification of syringin in ZP extract using high performance liquid chromatography (HPLC). Chromatogram of syringin standard (**a**) and ZP (**b**).

2. Cytotoxicity of ZP on HaCaT, HDFn, and RAW 264.7 cells

To evaluate the potential cytotoxicity of ZP, its effects on the viability of HaCaT, HDFn, and RAW 264.7 cells were first assessed prior to investigating its anti-inflammatory activity in lipopolysaccharide (LPS)-stimulated macrophages and its protective effects in periodontitis against experimental periodontal disease (EPD). HaCaT (human keratinocytes), HDFn (neonatal human dermal fibroblasts), and RAW 264.7 (murine macrophages) were obtained from the American Type Culture Collection (ATCC, Rockville, MD, USA). HaCaT and RAW 264.7 cells were cultured in Dulbecco's modified Eagle's medium (DMEM; HyClone Laboratories, Logan, UT, USA) with 10% fetal bovine serum (Lonza, Walkersville, MD, USA) and antibiotics, while HDFn cells were maintained in fibroblast basal medium (FBM; ATCC) supplemented with the FBM low-serum kit (ATCC). All cells were incubated at 37°C under a humidified 5% CO_2 atmosphere. For the viability assay, cells were seeded into 48-well plates at 0.5 × 10^4 cells per well, serum starved for 3 h, and then treated with ZP at concentration ranging from 0.001 to 10 mg/mL. After 24 h incubation, viable cells were stained with 3-(4,5-dimethylthiazol-2-yl)-2,5-diphenyltetrazolium bromide (MTT) solution (0.1 μg/mL) for 4 h. Formazan crystals were dissolved in dimethyl sulfoxide, and absorbance was measured at 570 nm using a microplate reader. Cell viability was expressed as relative to untreated controls. As a result, treatment with ZP did not cause any significant reduction in the viability of HaCaT, HDFn, or RAW 264.7 cells (Fig. S3).

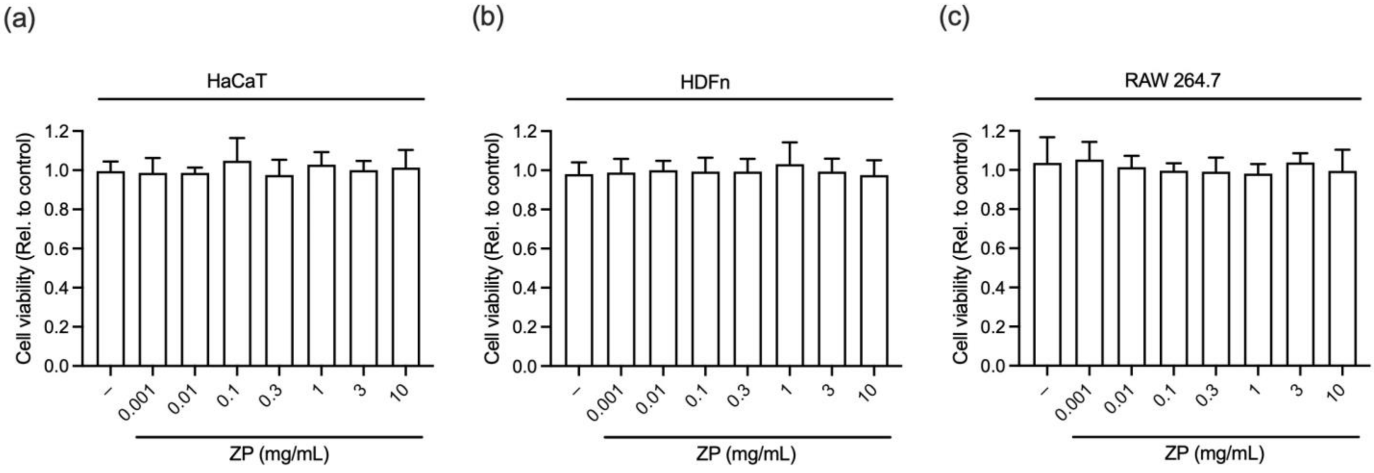

Figure S3. Effects of ZP on cell viability. (**a**) HaCaT (human keratinocytes), (**b**) HDFn (human neonatal dermal fibroblasts), and (**c**) RAW 264.7 (murine macrophages) were treated with ZP (0.001–10 mg/mL) for 24 h. Cell viability was assessed using the MTT assay, and results are expressed relative to untreated control.
